# Disparities in the regional, hospital and individual levels of antibiotic use in gallstone surgery in Sweden

**DOI:** 10.1186/s12893-017-0312-0

**Published:** 2017-12-06

**Authors:** Gona Jaafar, Bahman Darkahi, Lars Lindhagen, Gunnar Persson, Gabriel Sandblom

**Affiliations:** 1 Department of Clinical Sciences, Intervention and Technology (CLINTEC), Karolinska Institutet, Center for Digestive Diseases, Karolinska University Hospital, -141 86 Stockholm, SE Sweden; 2 Department of Surgery, Enköping Hospital, Kungsgatan 71, 74538 Enköping, Sweden; 3 Uppsala Clinical Research Centre, Dag Hammarskjölds väg 14B, 75237 Uppsala, Sweden; 4 Department of Surgery, Växjö Hospital, Strandvägen 8, 35185 Växjö, Sweden

**Keywords:** Cholecystectomy, Prophylactic antibiotic, Management programme, Cholecystitis

## Abstract

**Background:**

Antimicrobial resistance may be promoted by divergent routines and lack of conformity in antibiotic treatment, especially regarding the practice of antibiotic prophylaxis. The aim of the present study was to assess differences in gallstone surgery regarding antibiotic use in Sweden.

**Methods:**

The study was based on data from the Swedish Register for Gallstone Surgery and ERCP (GallRiks) 2005–2015. Funnel plots were used to test impact of grouping factors, including, hospital and surgeon and to identify units that deviated from the rest of the population.

**Results:**

After adjusting for cofounders including age, gender, ASA classification, indication for surgery, operation time, gallbladder perforation and emergency status, there were 0/21 (0%) at the regional level, 18/76 (24%) at the hospital level and 128/1038 (12%) at the surgeon level outside the 99.9% confidence interval (CI). The estimated median odds ratios were 1.13 (95% CI 1.00–1.31) at the regional level, 1.93 (95% CI 1.70–2.19) at the hospital level and 2.38 (95% CI 2.26–2.50) at the surgeon level.

**Conclusion:**

There are significant differences between hospitals and surgeons, but little or no differences between regions. These deviations confirm the lack of standardization in regards to prescription of antibiotic prophylaxis and the need more uniform routines regarding antibiotic usage. Randomized controlled trials and large population-based studies are necessary to assess assessing the effectiveness and safety of antibiotic prophylaxis in gallstone surgery.

## Background

In the absence of firm evidence regarding the effectiveness of antibiotic prophylaxis in gallstone surgery, there is a risk of non-stringent practice and arbitrary use of antibiotics. This may eventually lead to an increasing antimicrobial resistance. The increasing drug resistance is strongly promoted by divergent routines, especially regarding the attitude towards antibiotic prophylaxis [1–3].

Whereas there are several studies confirming the lack of benefit of antibiotic prophylaxis in routine laparoscopic cholecystectomy [4], there are, to our knowledge, no randomised controlled trials testing the benefits of antibiotic prophylaxis in complicated procedures such as open cholecystectomy, and procedures performed for cholecystitis or common bile duct stones.

The adherence to evidence-based routines regarding antibiotic prophylaxis in Greece [5], the Netherlands [6], the United States [3, 7], Japan [8] and Switzerland [9] have been studied previously. For complicated procedures, for which the benefits of antibiotic prophylaxis have not been evaluated in well-designed studies, the adherence to generally established routines is more difficult to determine. Although there are not sufficient studies to provide unequivocal guidelines on antibiotic prophylaxis and treatment of infectious complications in gallstone surgery, there are reasons to believe that conformity in prescription of antibiotics is one of the most important measures in the prevention of drug resistance [10, 11]. Uniform guidelines are thus important, even for patient groups and situations that have not been assessed in Level 1 studies.

The aim of the present study was to explore the use of antibiotic in gallstone surgery in Sweden and to determine, whether any deviation from the average could be explained by lack of conformity at the regional, hospital or surgeon level.

## Methods

This study is based on data from the Swedish Register for Gallstone Surgery and ERCP (GallRiks). GallRiks is a national register covering all open and laparoscopic surgery of the gallbladder performed in Sweden. It is continuously validated and has been shown to have 98% matches between the medical records and the database. Between 2006 and 2008, the register grew to cover more than 90% of Swedish surgical units of by the end of 2008 [12].

This study is based on all cholecystectomies registered in GallRiks 2005–2015. The variables used were gender, age, calendar year, method of approach, ASA classification, indication for surgery, operation time, accidental gallbladder perforation, antibiotic treatment and emergency status. Antibiotics were registered as none, prophylactic or therapeutic in GallRiks. In the present study, antibiotics given as prophylaxis (duration not intended to be longer than 24 h) and antibiotics given as therapy (duration longer than 24 h) were merged to one category since it was impossible to retrospectively determine the purpose of administrating antibiotics in each case. Choice of drug was also not registered. Only units where at least 25 cholecystectomies were performed were included in the analysis.

The following patients were excluded:

Patients for which data on antibiotic prophylaxis, age, gender, ASA, gallbladder perforation, emergency status or surgeon were missing.

Patients undergoing common bile duct exploration (post cholecystectomy status).

Patients undergoing cholecystectomy for indications other than gallstone disease.

Procedures with duration unknown or recorded as being longer than 24 h (assumed to be erroneous recordings).

Patients with indication vital indication was also removed.

Hospitals and surgeon with fewer patients than 25 were also excluded.

The number of excluded in each category is given in Table 1. The total numbers of excluded cases are 14,108 (12.5%).

**Table 1:** Assembling of the study cohort

Criterion	Number of remaining patients
Original data	113,209
Known antibiotics treatment	110,301
Known surgeon	109,681
Known ASA	109,664
Removed indication “undergoing other surgery”	109,196
Removed operation method “undergoing common bile duct exploration”	109,108
Known operation time ≤ 24 h	109,083
Known age	108,841
Known gender	108,839
Known gallbladder perforation	108,502
Known emergency status	107,925
Removed institutions with fewer than 25 patients	99,101

The study was approved by Stockholm ethical review board. All participants gave informed consent to inclusion. The study was conducted in accordance with the Helsinki declaration.

### Statistics

The funnel plots provide a graphical presentation of which units have antibiotic use outside the confidence intervals (the “funnel”), thus representing units deviating more from the general population than what would have been expected if there have been an underlying uniform approach and random variation at each level [13]. Three levels of grouping factors were defined: region, hospital and surgeon. The percentage of patients receiving antibiotic is plotted on the y axis and the total number of patients treated at the unit is plotted on the x axis. The 95% confidence intervals were defined as θ_0_ ± 1.96 x$\sqrt{p_0\left(1-{p}_0\right)/n}$, where*p*_0_ is the proportion of patients receiving antibiotics in the whole population and n is the number of patients in the unit.

We also constructed multi-level funnel plots, with the indicators derived from a Bayesian multilevel regression model, adjusted for other levels (e.g. region and hospital in the surgeon plot). As an example, imagine a hospital where all surgeons have high antibiotics prescription rates. This will be interpreted by the model as normal surgeons in a high-prescribing hospital. Therefore, the hospital will look extreme (outside the funnel), whereas the surgeons will not, in their respective funnel plots. In the multi-level plots, adjustments were also made for potential confounding factors i(age, gender, calendar year, ASA classification, indication for surgery, operation time, operation method, gallbladder perforation and emergency status). Patients with missing data were removed from the analysis (complete case-analysis). The underlying statistical method has been described in detail in a previous report [13].

## Results

Altogether 113,209 cholecystectomies were performed 2005–2015. From this group, 14,108 were excluded according to the exclusion criteria described above, leaving 99,101 patients in the study group. Baseline data of the study group are given in Table 2. There were relatively few patients during first two years When GallRiks established, 2005–2006, therefore these two years were pooled in order to get more balanced categories.

**Table 2:** Baseline data of the study group

Variable	No antibiotic *N* = 66,995	Antibiotic given *N* = 32,106	Combined *N* = 99,101
Age:			
≤ 40	23,114 (77.5%)	6720 (22.5%)	29,834
41–60	27,621 (70.3%)	11,671 (29.7%)	39,292
> 60	16,260 (54.2%)	13,715 (45.8%)	29,975
Gender:			
Male	18,775 (57.1%)	14,081 (42.9%)	32,856
Female	48,220 (72.8%)	18,025(27.2%)	66,245
ASA:			
1	38,134 (74.8%)	12,854 (25.2%)	50,988
> 1	28,861 (60.0%)	19,252 (40.0%)	48,113
Indication:			
Uncomplicated	49,722 (84.2%)	9331 (15.8%)	59,053
Complicated	17,273 (43.1%)	22,775 (56.9%)	40,048
Operation method:			
Laparoscopic	63,791 (73.9%)	22,534 (26.1%)	86,325
Open	3204 (25.1%)	9572 (74.9%)	12,776
Operation time (min):			
< 90	41,010 (81.4%)	9386 (18.6%)	50,396
≥ 90	25,985 (53.4%)	22,720 (46.6%)	48,705
Gallbladder perforation:			
No	50,878 (73.5%)	18,329 (26.5%)	69,207
Yes	16,117 (53.9%)	13,777(46.1%)	29,894
Emergency:			
Elective	56,475 (82.4%)	12,093 (17.6%)	68,568
Emergency	10,520 (34.5%)	20,013 (65.5%)	30,533
Year:			
2005–2006	3938 (60.8%)	2539 (39.2%)	6477
2007–2015	63,057 (68.1%)	29,567(31.9)	92,624

**Fig. 1:**
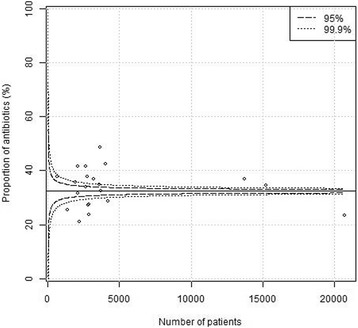
Plain funnel plot for regions. Outliers: 15/21 (71%)

**Fig. 2:**
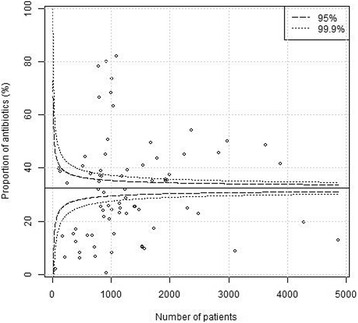
Plain funnel plot for hospitals. Outliers: 61/76 (80%)

**Fig. 3:**
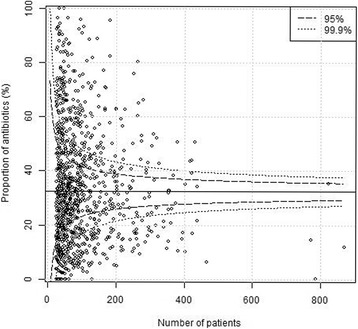
Plain funnel plot for Surgeons. Outliers: 400/1038 (39%)

**Fig. 4:**
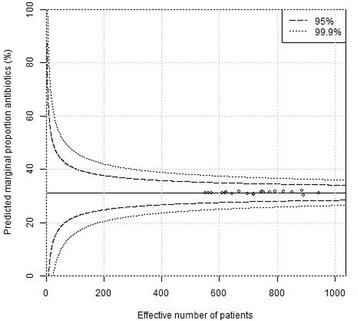
Covariate − adjusted multi − level funnel plot for regions. Outliers: 0/21 (0%)

**Fig. 5:**
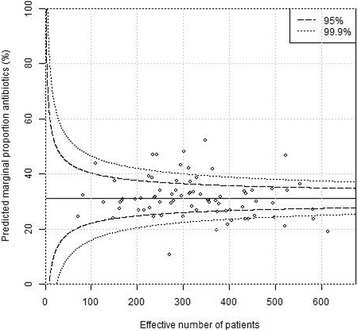
Covariate − adjusted multi − level funnel plot for hospitals. Outliers: 18/76 (24%)

**Fig. 6:**
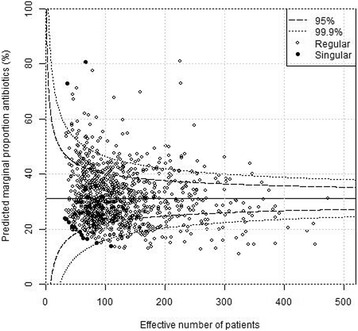
Covariate − adjusted multi − level funnel plot for surgeons. Outliers: 128/1038 (12%)

Plain funnel plots for regions, hospitals and surgeons are shown in Figs. 1, 2 and 3. At the region level, 15/21 (71%) of the regions were outside the 99.9% confidence intervals, at the hospital level 61/76 (80%) were outside the 99.9% confidence intervals, and at the surgeon level 400/1038(39%) were outside the 99.9% confidence intervals. I.e. there were large deviations from the national standard at the regional, local as well as surgeon levels in unadjusted comparisons. Multi-level funnel plots with adjustment for the above-mentioned confounding covariates, and level are shown in Figs. 4, 5 and 6. In these plots, all observations were within the 95% confidence intervals at the regional level. At the hospital level 18/76 (24%) were outside the 99.9% confidence intervals, and at the surgeon level 128/1038(12%) were outside the 99.9% confidence intervals. This indicates that there are standard routines at the regional level, but large deviations in routines at the hospital and surgeon levels.

In Table 3, estimates and credibility intervals for the random effect median odds ratios [14] achieved by fitting a mixed model to the data are given. The posterior mean, i.e. the outcome after adjustment for the covariates, is close to unity for region and high for hospital and region. This confirms that there are large differences between hospitals and surgeons, but not between regions.

## Discussion

This study shows a lack of conformity at the surgeon and hospital levels in Sweden, whereas there is no evidence of disparity at the regional level. How this affects the development of antibiotic resistance and the consequences for the individual patient is not fully understood. Nevertheless, good quality in health care is generally considered to include adherence to common routines.

Although resistant pathogens are not as widespread in Sweden as in other parts of Europe, standardized guidelines are necessary in order to prevent further spread across borders [15]. The disparities in practice at the hospital and surgeon levels probably reflect the evolvement of local routines that may be deeply rooted at each hospital, albeit without support from nationally accepted guidelines. The lack of uniform approach in the community may result in an uneven stimulation of drug resistance and a gradual spread of multiresistance bacteria. Judicious antibiotic utilization is an integral part of all stewardship programs and necessary to maximize clinical cure and minimize emergence of antimicrobial resistance [16]. As in many other situations where firm evidence is lacking, local traditions develop autonomously. These local traditions may give a misplaced feeling of following well-established principles in clinical decision-making, despite the absence of evidence-based support.

Standard common routines may be one of the best ways of reducing the risk for surgical site infections [7, 9]. By adopting the same routines at the surgeon and hospital levels, the risk for unequal distribution of antibiotic resistance is reduced. Local control of antibiotic prescription is probably crucial for preventing further spread of multi-resistant bacteria [17]. Furthermore, prudent use of antibiotics probably reduces the incidence of Clostridium difficile infections (CDI) and reduces drug costs.

A way of improving antibiotic prescribing practice is through antimicrobial stewardship programmes [18, 19]. The aim of such a programme is to monitor and guide the use of antibiotics. By benchmarking, hospitals and surgeons are given feedback on where they stand in comparison to other units. This is made possible by collaborative networks that evaluate the association between antimicrobial use and resistance. Antimicrobial stewardship programmes increase the understanding of how differences in antibiotic use at the hospital and surgeon levels affect the development of resistance. Implementation of an antimicrobial stewardship programme has been shown to lead to significant reductions in CDI rate, antibiotic use, and drug costs [16, 20]. Adherence to guidelines with regards to type, timing and duration of antibiotic prophylaxis was found to be 48% in one of the largest Italian hospitals [21]. This indicates that stewardship programs are necessary to achieve uniform routines. Active local feedback, repeated surveys and increasing awareness of antimicrobial resistance may improve compliance [22].

In a Cochrane review, it was concluded that several interventions aimed at reducing inappropriate use of antibiotics in hospitals might be effective in reducing the incidence of antimicrobial-resistant pathogens and Clostridium difficile-associated diarrhea [17]. The nature of such interventions varies, but effective programmes usually include continuous feedback reports, aimed at increasing awareness of the problem.

In 1994, the Swedish Strategic Programme for the Rational Use of Antimicrobial Agents and Surveillance of Resistance (STRAMA) was started [15, 23]. The goals of STRAMA are to preserve the effectiveness of currently available antibiotics, to work for better basic hygiene precautions, and more appropriate choice, dosage, and length of antibiotic treatment. However, even if STRAMA has played an important role in the reduction of antibiotic use, there is still room for improvement [22]. The present study shows that antibiotic routines for preventing surgical site infections in one of the most common procedures in Sweden, gallstone surgery, vary radically at the individual as well as the hospital level.

In a previous study, the incidence of infectious complications within thirty days after planned surgery for gallstones was found to be 4.3% [24]. Some of these infectious complications are very serious, so there is obviously a clear indication for antibiotic prophylaxis in selected cases. Nevertheless, routine administration of antibiotic without considering the role of antibiotics in healthcare in its greater perspective may have consequences that can only be perceived if each surgeon and hospital is continuously made aware of the local as well as national rates of antibiotic resistance.

The present study is limited by the difficulties in defining objective criteria for antibiotic prophylaxis and antibiotic use. Even if indication for surgery was included as covariate, there may have been circumstances related to the procedure that may have had an impact on the decision to give antibiotics, including previous or ongoing jaundice, concomitant ERC, degree of cholecystitis and co-morbidity. As many of these factors may have been unevenly distributed between the units, there may have been variations that could be explained by varying indications and not lack of conformity. Furthermore, no studies have been able to provide definite evidence for an association between local variations in antibiotic use and the ecology of microbial drug resistance, even if there are many reasons to assume such a relationship.

The data are assembled from GallRiks 2005–2015. Although there has been an ongoing debate regarding the use of antibiotics for prophylaxis in surgery since 2010, the routines have not changed much since then [13].

## Conclusion

In conclusion, the present study shows that the antibiotic usage routines are relatively uniform at the regional level, but vary at the hospital and surgeon levels in Sweden. This calls for standard antibiotic regimens and the development of national guidelines for use of antibiotic prophylaxis in gallstone surgery. The present study shows that uniform routines are necessary, although the level of evidence regarding antibiotic prophylaxis is insufficient. At present, there are ongoing randomized controlled trials and register-based studies with the aim of assessing the effectiveness and safety of AP in surgery for acute cholecystitis.

## Abbreviations

ASA: American Society of anesthesiologists
CDI: Clostridium difficile infections
CI: Confidence interval
GallRiks: Swedish Register for Gallstone Surgery and ERCP
STRAMA: The Swedish strategic programme for the Rational Use of Antimicrobial Agents and Surveillance of Resistance
